# HIV/HAART and the brain — what's going on?

**DOI:** 10.1186/1758-2652-13-S4-O35

**Published:** 2010-11-08

**Authors:** P Portegies

**Affiliations:** 1 Academic Medical Center, Deptartment of Neurology, University of Amsterdam, Amsterdam, Netherlands

## 

In the past 3-5 years cognitive impairment have been reported in 15-50% of long-term infected and long-term treated patients. Not full-blown HIV-dementia, but more subtle memory problems and slowness, difficulties in concentration, planning, and multitasking are the characteristic complaints. Even in patients who are systemically well-controlled these problems do occur.

So what is going on after so many years of apparently controlled HIV-infection in the CNS? How severe is the problem? Is it HIV-driven? Was HIV-infection well under control in all patients without complaints or have we missed ongoing replication in the brain? Has this ongoing replication led to chronic immune-activation and progressive damage to the brain? And what is the role of co-morbidity in an HIV-infected population that has entered their fifties and sixties. Do the "normal", non-HIV-related aging of the brain with vascular white matter abnormalities cause additional damage to the brain? Do we see cognitive problems, similar to the patterns that we see in Alzheimer's disease or sepsis where inflammatory changes in the brain seem to play an important role? Is the process of normal aging accelerated in HIV-infection?

Chronic HIV-driven inflammation in an aging brain could be the cause of what we see clinically today. It is likely that HIV-replication in the brain is not the only factor. Better CNS-penetrating drugs will not be the only answer. Clinical characteristics, diagnostic tools (new imaging techniques and CSF-analysis), course and prognosis, possible interventions, antiretroviral and adjunctive therapies will be discussed. The research agenda for HIV-neurology is filled for the years ahead.

